# Supplementary material for: A Flexible Piezocapacitive Pressure Sensor with Microsphere-Array Electrodes
Source: Nanomaterials (Basel). 2023 May 23;13(11):1702. doi: 10.3390/nano13111702 (PMC10254135; doi:10.3390/nano13111702)
Supplement: Supplementary file 1 [file nanomaterials-13-01702-s001.zip › nanomaterials-2372085-supplementary.pdf]

Supporting Information

A Flexible Piezocapacitive Pressure Sensor with Microsphere Array Electrodes

Shu Ying[1,†], Jiean Li[1,†], Jinrong Huang[1,*], Jiahan Zhang[1], Jing Zhang[1], Yongchang Jiang[1], Xidi Sun[1,*], Lijia Pan[1,*], Yi Shi[1]

[1] Collaborative Innovation Center of Advanced Microstructures, School of Electronic Science and Engineering, Nanjing University, Nanjing 210093, China

[*] Correspondence: xidisun@smail.nju.edu.cn (X.S.); jinronghuang@smail.nju.edu.cn (J.H.); ljpan@nju.edu.cn (L. P.)

[†] These authors contributed equally to this work.

Contents

A: Preparation of piezocapacitive pressure sensor

B: Cyclic stability test

C: Mechanism for enhancing the performance of microsphere electrode based sensors

A:Preparation of piezocapacitive pressure sensor

In our experiments, the glass pieces were placed in a glass petri dish, poured into acetone solution, placed in an ultrasonic cleaner, and ultrasonicated for 15 min. and then placed in a petri dish with ethanol solution and ultrasonicated for 10 min to remove the oil and impurities on the surface of the glass pieces. The cleaned glass slides were placed in a UV-ozone cleaner and hydrophilically treated for 20 min to make the surface with good hydrophilicity. 1 ml of PS microsphere mixture was removed with a pipette, placed in a beaker, and 5 ml of ethanol solution was added and sonicated in an ultrasonic cleaner for 10 min, at which time the PS microspheres were uniformly dispersed in the ethanol solution. Using the self-assembly method, the proportioned solution was evenly added dropwise to the glass sheet and left to self-assemble into a uniform monolayer film at room temperature. When the ethanol evaporates, the PS microspheres are uniformly distributed on the surface of the glass sheet, and a monolayer of PS microspheres is obtained.

B: Cyclic stability test

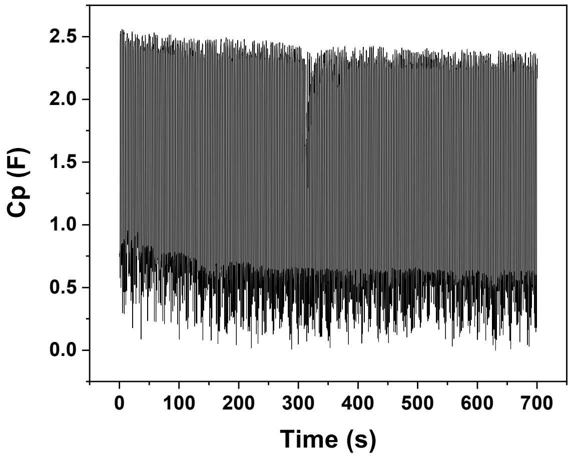

Figure S1. Finger bending cycle test.

C: Mechanism for enhancing the performance of microsphere electrode based sensors

Conventional microstructured devices have a conductive layer on the flat backside with a microstructured PDMS. In this design, the total capacitance is approximately contributed by two capacitors connected in series: an elastomer-based capacitor, which shows a relatively small variation in compression. Thus, the normalized capacitance variation can be expressed as：

$$\frac{\Delta C_{Conventional}}{C_0} = \frac{\varepsilon_{PDMS}\Delta d_{Air}+\varepsilon_{Air}\Delta d_{PDMS}}{d'_{Air}\varepsilon_{PDMS}+d'_{PDMS}\varepsilon_{Air}} \approx \frac{\varepsilon_{PDMS}\Delta d_{Air}}{d'_{Air}\varepsilon_{PDMS}+d'_{Air}\varepsilon_{Air}} \quad (1)$$

where εAir (=1.0) and εPDMS (~3.0) are the dielectric constants of air and PDMS, respectively, Δd_{Air} and Δd_{PDMS} are the changes in thickness of air and PDMS, individually, and d'_{Air} and d'_{PDMS} are the final thicknesses of air and PDMS under applied pressure[1]. In this conventional structure, the thinner the elastomer dielectric, the higher the sensitivity and signal-to-noise ratio that can be achieved by the sensor. However, aggressively reducing the thickness of the elastomer while maintaining the air gap of the microstructure is a considerable challenge, which limits the normalized capacitance variation and sensitivity of such devices. However, attaching electrodes directly to the microstructure allows the capacitance to be dominated by the air gap component as the main contribution, thus reducing the influence of the elastomer electrolyte and thus greatly improving the sensitivity.

References

1. Huang, Y.-C.; Liu, Y.; Ma, C.; Cheng, H.-C.; He, Q.; Wu, H.; Wang, C.; Lin, C.-Y.; Huang, Y.; Duan, X., Sensitive pressure sensors based on conductive microstructured air-gap gates and two-dimensional semiconductor transistors. *Nature Electronics* **2020,** *3* (1), 59-69.
